# Is adjunctive naturopathy associated with improved glycaemic control and a reduction in need for medications among type 2 Diabetes patients? A prospective cohort study from India

**DOI:** 10.1186/s12906-016-1264-0

**Published:** 2016-08-17

**Authors:** Srinivas Bairy, Ajay M. V. Kumar, MSN Raju, Shanta Achanta, Balaji Naik, Jaya P. Tripathy, Rony Zachariah

**Affiliations:** 1 Mantena Satyanarayana Raju Arogyalayam, Vijayawada, Andhra Pradesh India; 2 International Union Against Tuberculosis and Lung Disease (The Union), South-East Asia Regional Office, C-6, Qutub Institutional Area, New Delhi, 110016 India; 3 WHO Country Office for India, New Delhi, India; 4 School of Public Health, PGIMER, Chandigarh, India; 5 Medecins Sans Frontieres, Brussels Operational centre, Luxembourg, Luxembourg

**Keywords:** Complementary medicine, reversal of diabetes, SORT-IT, Operational research

## Abstract

**Background:**

With an estimated 65 million Diabetes Mellitus (DM) patients, India ranks second in the world in terms of DM burden. The emphasis of current medical practice has been on pharmacotherapy but, despite the best combination therapies, acheiving glycaemic control (reduction of blood sugar to desirable levels) is a challenge. ‘Integrated Naturopathy and Yoga’(INY) is an alternative system of medicine that lays emphasis on the role of diet and physical exercise. We assessed the short term effect of INY as an adjunct to pharmacotherapy on glycaemic control among type 2 DM patients.

**Methods:**

In this prospective cohort study with a 3 month follow-up, DM patients consecutively admitted to a hospital in India from May-October 2014 for either 15 or 30 days were offered INY - a package of vegetarian diet with no added oil, sugar and salt, yoga-based exercise, patient counselling and rest. A ‘favourable outcome’ was defined as glycaemic control (glycosylated hemoglobin (HbA_1c_) < 7 % or absolute reduction by 1 %) along with at least 50 % reduction in antidiabetes medication at 3 months relative to baseline. Compliance to diet was scored by self-report on a scale of 0–10 and categorized into poor (0–5), moderate (6–8) and excellent (9–10).

**Results:**

Of 101 patients with 3-month follow-up data, 65(65 %) achieved a favourable outcome – with 19(19 %) stopping medication while sustaining glycemic control. Factors associated with favourable outcome were baseline HbA_1c_ and compliance to diet, which showed a significant linear relationship with mean HbA_1c_ reductions of 0.4 %, 1.1 % and 1.7 % in relation to poor, moderate and excellent dietary compliance respectively.

**Conclusion:**

INY, adjunctive to pharmacotherapy, was associated with a significant beneficial effect on glycaemic control and reduced the overall need for antidiabetes medications. These early results are promising. Further studies with long-term follow-up and using more rigorous randomized controlled trial designs are needed.

**Electronic supplementary material:**

The online version of this article (doi:10.1186/s12906-016-1264-0) contains supplementary material, which is available to authorized users.

## Background

Diabetes Mellitus (DM) is a chronic condition characterized by increased levels of glucose in the blood and if uncontrolled, can lead to multiple complications including ischemic heart disease, stroke, blindness, limb amputations as well as dysfunction of nerves and kidneys. The most common form of DM is type 2 which constitutes more than 90 % of cases. More than 80 % of DM patients now live in Low and Middle Income Countries (LMIC) and the disease is being propelled by unhealthy diets and sedentary lifestyles. The long-held myth that DM affects only affluent countries is thus no longer true [1].

In India, there are an estimated 65 million DM patients and another 77 million in a pre-diabetic state - India now ranks second in the world (next to China) in terms of DM burden [1, 2]. The emphasis of current medical practice has been on reducing the blood sugar using drugs (akin to treating the symptoms) rather than addressing the root causes – which are related to faulty dietary habits, lack of exercise and lifestyle [3]. Further, the drugs used have several known and unknown side effects and despite combination therapies, glycaemic control is challenging to achieve and the disease often progresses to complications giving value to the adage, ‘once a diabetic, always a diabetic’ [4, 5]. Offering patients a structured and empowering environment to implement intense lifestyle interventions, are urgently required to foster adjunctive measures including lifestyle changes.

‘Naturopathy and Yoga’ is an alternative form of medicine that lays emphasis on the role of diet and physical exercise in health promotion and reversal of disease. As part of the general philosophy of ‘*aligning oneself in tune with nature’*, naturopathy recommends intake of large quantities of water (about 4–5 litres per day), vegetarian food in its natural form including use of sprouted high-protein pulses, fruits and vegetables, unrefined whole-grains, with no added oil, sugar and salt, yoga-based exercise and rest. Several studies have shown beneficial effects of individual lifestyle interventions (including a controlled diet), weight loss and exercise on DM management outcomes [6–11]. However, none have assessed the effect of an integrated management approach in achieving glycaemic control and reducing the reliance on anti-diabetes medication.

The ‘Mantena Satyanarayana Raju Arogyalayam’ (MSRA) hospital, situated in South India offers an ‘Integrated approach of Naturopathy and Yoga’ (INY) in addition to pharmacotherapy. There is anecdotal evidence that some DM patients following INY do not need anti-diabetes medications anymore for maintaining their glycemic control.

To date, there has been no systematic assessment of the effect of INY, when used as adjunct to pharmacotherapy, on glycaemic control among DM patients. Such evidence would be useful in contemplating the role INY could play within mainstream DM care. As a preliminary step, we thus aimed to assess the short term effect of INY as an adjunct to pharmacotherapy, in achieving glycaemic control. The specific objectives were to determine among a cohort of type 2 DM patients enrolled into INY a) the number (and proportion) who achieved favourable outcomes (glycaemic control achieved along with at-least 50 % reduction of anti-diabetic medication at 3 months after enrolment) and b) socio-demographic and clinical factors associated with favourable outcomes.

## Methods

### Ethics

Approval was received from the Ethics Advisory Group of the International Union Against Tuberculosis and Lung Disease, Paris, France and the Institutional Ethics Committee of MSRA, Guntur, India. Written informed consent was obtained from all the study participants.

### Study design and period

This was a prospective cohort study involving follow-up of type 2 DM patients for 3 months and was conducted between March and December 2014.

### Study population

All known cases of type 2 DM who were on anti-diabetes medications (for at-least 1 year) and consecutively admitted to MSRA hospital from May to August 2014 were included. The following sub-groups were excluded: children (<18 years), pregnant women, patients with diabetes related complications related to kidney, heart, eye, nervous system and any communicable disease at the time of admission to MSRA. We did not exclude any patients based on blood sugar levels at baseline.

### Study setting

#### The Mantena Satyanarayana Raju Arogyalayam’ (MSRA) hospital

MSRA Hospital is a 500-bedded, fully residential naturopathy institute located in Amaravathi city of Andhra Pradesh, a south Indian state (population 50 million). It is the largest naturopathy institute in India, spread across an area of 18 acres in natural, pollution-free green surroundings. Patients with a variety of chronic diseases including diabetes mellitus, hypertension and cardiovascular diseases seek care at the institute. People also seek care at the institute for simple weight reduction and general health promotion. There are ten departments managed by around 300 trained staff including one general physician, 14 naturopathy doctors, and 120 paramedical workers. The institute is equipped with a yoga hall, a laboratory, emergency medical services, a physiotherapy unit and facilities for naturopathy treatments.

#### Medical and INY management of DM

On admission, each DM patient is assessed by a qualified medical doctor and his/her clinical details are recorded in a treatment card. The pharmacological management (including decisions on the changing the doses of anti-diabetic medications) is done by a diabetologist based on clinical assessment (hypoglycemic symptoms) and laboratory parameters (evidence of normal FBS and PPBS).

Individuals have the option of staying for either 15 or 30 days in the institution when they learn and practice INY under supervision of qualified doctors. The integrated INY package provided to the patients included diet, exercise, rest and relaxation and patient education and is shown in Table 1.

**Table 1:** Package of Integrated Naturopathy and Yoga provided at 'Manthena Sathyanarayana Raju Arogyalayam' hospital for management of Diabetes mellitus in Andhra Pradesh, India, May-August, 2014

Component	Description
Diet	A special package of diet is designed for DM patients admitted in MSRA containing low glycaemic-index, fibre-rich, plant-based diet containing whole grains, legumes, vegetables and fruits which are high in protein and no added salt, oil and sugar. Nearly two-thirds of the diet is constituted by the foods in their natural form (not cooked) which includes high-quality, protein-rich sprouted pulses and fruits and vegetable salads. Indian breads prepared out of whole-wheat flour are provided instead of steamed rice as a source of carbohydrate. Special care is taken to prepare tasty, low-calorie recipes and flavoured with locally available herbs without the use of additional salt, spices and oil. Contrary to other reported dietary interventions in the published literature, there was no restriction in the quantity of the food consumed by the patients, who are encouraged to eat as much as they want. A principle of ‘early dinner’ (before 7:00 pm) is followed. This is in tune with the behaviour of all diurnal animals in nature and is intended to provide adequate rest to the body, especially the organs involved in the digestion of food. The meals (breakfast, lunch and dinner) are provided at pre-specified times of the day to ensure that the body gets into a natural rhythm of metabolism. Patients are also taught the methods of preparing the recipes in ‘daily cooking classes’ (1 hour) to enable them to prepare their meals once they return home.
Exercise	Regular exercise (two times a day) in the form of Yogic exercises and Pranayama (breathing exercises) are taught to the patients who practice under the supervision of qualified yoga teachers. Emphasis is given on ‘Asanas’ (yogic postures) which have an effect on weight reduction (especially reducing the abdominal fat stores) and stimulation of pancreas to produce insulin. In addition, patients are encouraged to do aerobic exercises like walking, swimming or boat-pedalling in the river.
Rest and Relaxation	Several measures are taken to ensure that patients are adequately rested and live a stress-free life in the institute. In addition to the relaxing and quiet environment of the MSRA, daily lectures are provided about stress and methods of addressing them. Interested patients are also offered an opportunity to learn meditation and practice under supervision of experts. Naturopathy advocates “Therapeutic Fasting” using honey, lemon and water which allows the body to rest completely and heal itself. Patients who are able to completely stop anti-DM medications and whose blood sugar is under control are offered an opportunity to fast for 1–3 days. Patients are carefully monitored during this process for their blood sugar and electrolytes. In addition, several treatments such as various types of mud/steam bath and body massages are administered to relieve pain and relieve stress.
Patient education	Naturopathy emphasizes on self-responsibility of one’s own health. A minimum of 15 days of stay in the institution is needed to understand the rationale of the intervention and for motivating the patients to practice on their own. The emphasis is on patient empowerment to promote a sense of self-responsibility after going back to their day-to-day lives and in the long run. There are daily lectures and discussion in the evening (60 minutes) to provide the patients an understanding of the principles of naturopathy. There are also daily morning lectures (60 minutes) on personality development, stress management and meditation. Videos and books about these principles are also made available to patients. In addition, patients are counselled one-to-one by the doctors every day.

After discharge, they are advised to apply the principles and practice of INY in their routine lives. At baseline, height, weight, blood pressure, fasting plasma glucose (FPG), post prandial plasma glucose (PPPG), glycosylated haemoglobin (HbA_1c_), plasma sodium and potassium levels and the details of various medications taken by the patient are recorded. These parameters are repeated as per individual patient needs, at the time of discharge and at 3 months from admission. To ensure standardization and quality control of laboratory procedures, all investigations were conducted at a quality-assured laboratory called ‘Thyrocare’ (http://www.thyrocare.com/) situated in Navi Mumbai, India. Arrangements were made through Thyrocare blood collection centres (available throughout the country) to collect and transport blood specimens. While patients had to pay for stay, food and other incidentals, costs of laboratory investigations were covered by the hospital.

### Data collection, definition of a favorable outcome and analysis

A pre-tested, data collection format was used to collect information related to the study objectives. Every patient was followed up over telephone on a monthly basis for 3 months. Phone calls were made by specially trained, independent interviewers. Study participants graded themselves on a scale of 0–10 (“0” for non compliance and ‘10’ for full compliance) for compliance to recommended dietary advice and practice of yoga. There were three questions asked on diet: How do you grade yourself on a scale of 0–10 about eating i) sprouted pulses for breakfast? ii) diet free of added sugar, salt and oil? iii) having dinner before 7 pm? An average of the scores was taken and patients were divided into three groups: poor (0–5), moderate (6–8) and excellent (9–10). An appointment at home was made (after consent) to collect blood samples for laboratory analysis.

*Definition of a ‘favourable outcome’:* Achievement of glycaemic control along with at-least 50 % reduction in dose of anti-diabetes medication at 3 months relative to baseline constituted a favourable outcome. Glycaemic control was further defined based on HbA_1c_ – for those with baseline HbA_1c_ < 7 %, maintaining it below 7 % at 3 months was considered glycaemic control and for those with baseline HbA_1c_ ≥ 7 %, this was defined as reducing HbA1c at 3 months to <7 % or an absolute reduction by 1 % relative to baseline. The cut-off of 1 % was chosen based on previous studies indicating that a drop of 1 % in HbA1c is associated with favourable long-term cardiovascular outcomes [12]. When the patient was on multiple drugs, the reduction in dose was assessed for each drug and averaged. Any increase in dose of existing drugs and addition of a new drug was considered ‘unfavourable’.

Data entry was done using EpiData Entry software (version 3.1, EpiData Association, Odense, Denmark). Clean data along with the codebook after removing personal identifiers have been provided in additional files 1 and 2. Analysis was performed using EpiData Analysis software (version 2.2.2.182) and STATA 12.1 (Stata Corporation, Texas 77845, USA). Differences in proportions were calculated using the chi-square test and trends were assessed using the chi-square for linear trend. The unpaired t-test (or kruskal wallis test) or paired t-test was used to assess difference in means, as appropriate. Factors independently associated with a favourable outcome were identified using multivariate logistic regression analysis. The level of significance was set at *P* ≤ 0.05.

## Results

Of 223 patients enrolled, 101 could be followed up at home at 3 months. The characteristics of individuals who could and could not be assessed were similar (Table 2). The reasons for not being able to reach 122 individuals at home included unavailability of patients at the time of scheduled contact (due to engagement in social commitments, job and/or travel) and long distances from the hospital making logistics for the follow-up team difficult.

**Table 2:** Demographic and clinical characteristics of patients who could and could not be assessed at 3 months following admission to Manthena Sathyanarayana Raju Arogyalayam (MSRA) hospital for management of Diabetes mellitus using integrated Naturopathy and Yoga in Andhra Pradesh, India, May-August, 2014

Characteristics	Assessed	Not assessed	*P*-value
Total	101	122	
Male – Number (%)	55 (55)	67 (55)	0.94
Mean age (95 % CI)	55 (53–56)	52 (50–54)	0.11*
Mean duration of DM (95 % CI)	7.6 (6–9)	7.2 (6–8)	0.61
Mean FBS (95 % CI)	149 (137–160)	148 (138–159)	0.97
Mean PPBS (95 % CI)	188 (174–202)	185 (173–198)	0.78
Mean HbA_1c_ (95 % CI)	8.2 (7.8–8.6)	8.3 (8.0–8.6)	0.71
On oral drugs – Number (%)	86 (85)	104 (85)	0.98

*DM* Diabetes Mellitus, *CI* Confidence interval, *HbA*_*1c*_ Glycosylated hemoglobin, *FBS* Fasting blood sugar, *PPBS* Post Prandial Blood Sugar, *CI* Confidence Intervals

*Kruskal Wallis test was used

Table 3 shows the socio-demographic and clinical characteristics of those admitted and assessed at 3 months. Their median age was 55 years (Interquartile range (IQR), 49–62) and median duration of DM was 6 years (IQR, 2.5–10). There were 40(40 %) patients who had associated hypertension. About 85 % received oral hypoglycemic drugs while the rest were on insulin injections. Administered drugs included metformin, glimeperide, gliclazide, voglibose, sitagliptin, pioglitazone and insulin. Overall 31(30 %) patients received one drug, 45(45 %) received two drugs, 18(18 %) received three drugs and 7(7 %) received four drugs at baseline.

**Table 3:** Socio-demographic and clinical characteristics of individuals admitted to Manthena Sathyanarayana Raju Arogyalayam (MSRA) hospital for management of Diabetes mellitus using integrated Naturopathy and Yoga in Andhra Pradesh, India, May-August, 2014

Category	Sub-category	N (%)
Total		101 (100)
Age (years)	<60	66 (65)
	≥60	35 (35)
Sex	Male	55 (55)
	Female	46 (45)
Occupation	Housewife	40 (40)
	Retired	20 (20)
	Self-employed (business)	19 (19)
	Government employee	12 (12)
	Private employee	7 (7)
	Unemployed	3 (3)
Education (years in school)	<12	50 (50)
	≥12	51 (51)
Associated Hypertension	No	61 (60)
	Yes	40 (40)
Family income/annum	<4000 USD	52 (51)
	≥4000 USD	49 (49)
History of tobacco smoking	Current smoker	4 (4)
	Past smoker	5 (5)
	Never smoker	92 (91)
History of alcohol intake	Current drinker	8 (8)
	Past drinker	6 (6)
	Never drank	87 (86)
Duration of diabetes (Years)	1–2	25 (25)
	3–5	24 (24)
	6–9	19 (19)
	≥10	33 (32)
Diabetic medication	Oral	86 (85)
	Insulin alone or with oral	15 (15)

*USD* United States Dollar; Current smoker: history of smoking tobacco in the past 1 month

Current drinker: history of consuming alcohol in the past 1 month

**Fig. 1:**
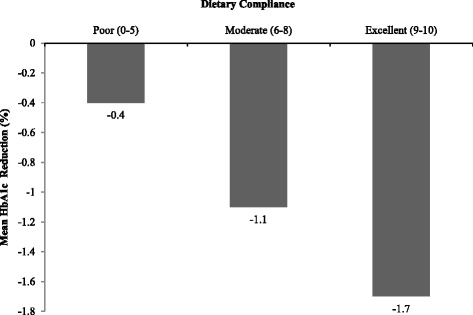
Dose response relationship between self-reported dietary compliance and mean reduction in HbA_1c_ at 3 months relative to baseline among type 2 DM patients admitted for Integrated Naturopathy and Yoga management to the Manthena Sathyanarayana Raju Arogyalayam (MSRA) hospital, Andhra Pradesh, India, May-August, 2014

Glycaemic control parameters measured at baseline and at 3 months after INY, showed significant mean reductions in levels of HbA1C, FBS and PPBS - respectively 0.9 %, 39 mg/dl and 36 mg/dl (*P* < 0.001, Table 4). This effect was more pronounced among patients with a baseline HbA1c of ≥7 %, with mean reductions of 1.2 %, 53 mg/dl and 53 mg/dl for HbA1c, FBS and PPBS respectively. There was a dose-response relationship between compliance to dietary practices and HbA1c at 3 months with mean reductions of 0.4 %, 1.1 % and 1.7 % among those with poor, moderate and excellent compliance to dietary practices respectively (Fig. 1).

**Table 4:** Effect of Integrated Naturopathy and Yoga management on glycaemic control (baseline and 3 months) among type 2 Diabetes patients admitted to Manthena Sathyanarayana Raju Arogyalayam (MSRA) hospital, Andhra Pradesh, India, May-August, 2014

Glycaemic control parameter	Mean baseline level	Mean level at 3 months	Mean difference (95 % CI)	*p*-value
HbA_1C_ (%)	8.2	7.3	0.9 (0.6–1.2)	<0.001
FBS (mg/dl)	149	109	39 (27–52)	<0.001
PPBS (mg/dl)	188	152	36 (23–49)	<0.001

*CI* Confidence interval, *HbA1C* Glycosylated hemoglobin, *FBS* Fasting blood sugar, *PPBS* Post Prandial Blood Sugar, *mg/dl* milligram per deciliter

Of the 101 individuals assessed at 3 months, 65 (65 %) had a ‘favourable outcome’ of whom, 19 stopped medication completely but sustained their HbA1c level below 7 %. Even among the 36 patients who did not meet the definition of favourable outcome, 23 had varying reductions of HbA1c (but less than 1 %) and tapering of medications (but less than 50 %).

Significant factor associated with a favourable outcome after adjusting for age, sex, education, baseline BMI, associated hypertension, compliance to yoga, income, duration of stay, baseline HbA_1c_ and duration of DM was compliance to advised diet (Table 5).

**Table 5:** Factors associated with a favourable management outcome (at 3 months) among patients admitted for Integrated Naturopathy and Yoga management to the Manthena Sathyanarayana Raju Arogyalayam (MSRA) hospital, Andhra Pradesh, India, May-August, 2014

Category	Subcategory	Total	Favourable outcome n (%)	aOR (95 % CI)	*p*-value
Total		101	65		
Age group (years)	<60	66	44 (67)	1.0	
	≥60	35	22 (63)	0.5 (0.1–1.7)	0.29
Sex	Male	55	40 (73)	1.0	
	Female	46	26 (57)	0.4 (0.1–1.4)	0.15
Education (years in school)	<12	50	30 (60)	0.9 (0.3–2.7)	0.87
	≥12	51	36 (71)	1.0	
Hypertension	No	61	37 (61)	1.0	
	Yes	40	29 (73)	1.5 (0.5–4.4)	0.50
Family income/annum	<4000 USD	52	32 (62)	1.0	
	≥4000 USD	49	34 (69)	1.4 (0.5–4.2)	0.52
Duration of diabetes (Years)	1–2	25	20 (80)	1.0	
	3–5	24	13 (54)	0.2 (0.1–1.2)	0.07
	6–9	19	11 (58)	0.3 (0.1–1.7)	0.16
	≥10	33	22 (67)	0.6 (0.1–4.4)	0.62
Diabetic medication	Oral	86	57 (66)	1.0	
	Insulin	15	9 (60)	0.9 (0.2–4.2)	0.94
BMI (kg/m^2^)	<25	23	15 (65)	1.5 (0.4–6.6)	0.62
	25.0–29.9	43	30 (70)	1.8 (0.4–8.7)	0.45
	30.0 and above	34	20 (59)	1.0	
Duration of stay	15 days	81	53 (66)	1.0	
	30 days	20	13 (65)	0.8 (0.2–3.9)	0.81
Baseline HbA_1c_	<7 %	28	23 (82)	1.0	
	≥7 %	73	43 (59)	0.4 (0.1–1.4)	0.14
Compliance to advised diet^a^	Poor	19	7 (37)	1.0	
	Moderate	32	19 (59)	3.8 (0.7–20.6)	0.12
	Excellent	49	40 (82)	16.5 (3.0–91.0)	0.001^b^
Compliance to Yoga^a^	Poor	27	16 (59)	1.0	
	Moderate	23	17 (74)	1.2 (0.2–7.0)	0.84
	Excellent	50	33 (66)	0.5 (0.1–2.1)	0.32

*USD* United States Dollars, *BMI* Body Mass Index, *HbA*_*1c*_ Glycosylated hemoglobin, *aOR* adjusted Odds Ratio, *CI* Confidence Intervals

^a^Poor: score of 0–5; Moderate: score of 6–8; Excellent: score of 9–10

^b^Chi-square for linear trend = 12.1, *P* <0.001

## Discussion

This is the first study from India showing the adjunctive and beneficial association of INY on glycemic control among DM patients. INY, when provided as adjunct to pharmacotherapy, was associated with not only achieving glycemic control in two-thirds of patients but also in reducing by half (or more) their need for antidiabetes medication. In about two-in-ten patients, DM medications were stopped and glycemic control was still sustained. These changes are impressive when considering the relatively short follow-up time span (3 months) and involving a cohort with a median duration of DM of 6 years. Furthermore, these findings might indicate the beginning of the process of reversal of DM and brings cautious early optimism to counter the adage “once a diabetic always a diabetic”. We would like to caution though, that these are early results and we need to follow-up the cohort for a longer duration to see if these changes will be sustained and lead to overall reductions in cardiovascular mortality.

The average reduction in HbA1c was high at 0.9 % which is comparable to results in other studies showing the effect of different dietary approaches on glycaemic control [6, 7, 13]. Such a significant effect could be related to the integrated approach implemented within a structured and enabling environment and involving multiple interventions that fostered lifestyle changes, each of which have been shown to be beneficial in previous studies [6, 7, 14–16].

The proportion of favourable outcomes were similar across the subgroups of patients and the only two associated significant factors were baseline HbA1c and compliance to diet. This indicates that INY could be equally effective irrespective of duration of DM, type of medications, age, sex, education, income, or hypertension. Compliance to diet showed a highly significant dose response relationship underlining its importance in achieving glycemic control. This maybe attributed to the many unique features of the naturopathy diet in comparison with other recommended dietary approaches - natural foods with no added oil, salt and sugar and no restriction in quantity of food intake.

The exact mechanism of action of INY in improving glycaemic control is not known. We speculate a few possible mechanisms. Previous studies have shown that weight loss is associated with glycaemic control [9]. A couple of studies on reversal of DM have been mostly among obese individuals who underwent surgical interventions (like gastric bypass surgery and other bariatric procedures) and was associated with sudden weight loss [17]. Some studies have associated low-fat vegan diets to reduction of intra myocelluar lipids, pancreatic and hepatic triacyl glycerols thus reversing insulin resistance and restoring beta cell function [9, 18, 19]. Given that INY diet does not use any added oils for cooking, this could be a possible mechanism. This merits further study.

There were several strengths to the study. First, INY was provided in a structured and enabling environment under supervision of qualified doctors and counsellors. This might have fostered lifestyle behaviour change - one of the most challenging things to achieve. Second, outcome was assessed using HbA_1c_ which provides the most reliable assessment of glycaemic control as it is less susceptible to temporary swings in blood sugar. Third, collection and analysis of laboratory tests were standardized and quality-controlled. Fourth, we were rather strict in defining the principal outcome measure which included not only achievement of glycemic control (which is the standard practice) but also a simulataneous and substantial reduction of DM medication by at least half from baseline doses. If such a strict threshold was not used and considered any reduction in medication and/or any reduction in HbA_1c_ as favourable, then the proportion with favourable outcome would have been even higher.

This study has several implications for policy and practice. First, it highlights the important adjunctive role for alternative medicine in improving glycaemic control and the quality of life [13, 20, 21]. There are also likely cost saving benefits for patients and health systems – direct costs arising from drugs and management of complications and indirect costs from loss of productivity. Given the massive economic burden posed by DM on global health care expenditure [1], approaches such as INY need to be considered. Future studies should examine the cost-effectiveness of this approach.

Second, even though the value of lifestyle interventions are acknowledged widely, in practice they prove to be difficult to implement. In this light, INY brings a sense of optimism with nearly 80 % of patients showing moderate to excellent dietary compliance. While the role of pharmacotherapy remains important, this study emphasizes the need for lifestyle changes. Clinicians often feel ill-informed about providing lifestyle advice and may lack time and capacity to take on this role [22, 23]. A possible way forward is to make DM care interdisciplinary with specialists from various systems of medicine working together as a team – allopathic doctors taking care of pharmacological management while delegating the responsibility of intense lifestyle interventions to alternative systems of medicine. The latter are often better versed to handle such aspects [21, 24].

We had some study limitations. Nearly half of the cohort could not be contacted for follow-up investigation at 3 months and this might have introduced responder bias. However, the baseline characteristics of responders and non-responders were similar especially duration of DM and baseline values of HbA_1c_, FBG and PPBG, possibly suggesting that the results can be generalised to the entire cohort. Also the patients had to be followed up at home and those excluded were due to logistic constraints in reaching them and does not reflect health seeking behaviour. Importantly this assessment involved a short follow-up period of 3 months and one wonders if patients will continue to be compliant to INY in the longer term and if favorable effects can be sustained. This is currently being studied in our setting and cohort studies with longer-term follow-up will enlighten us. Another limitation relates to the fact that this was a single arm study with no comparison group. Ideally a Randomized Controlled Trial would have provided the most robust evidence possible. However, since the patients coming to MSRA hospital want to undergo lifestyle intervention on their self-will, it will be challenging and arguably unethical to implement a randomized controlled trial. Finally, it is probable that patients who visited our hospital and participated in the study were more likely to be health-conscious and INY compliant than the general population. A qualitative study has shown that patients on DM medication get tired of side effects and long-term DM complications making them opt for naturopathy [21]. Alternative approaches such as INY may thus have higher patient acceptability in the longer run [13].

## Conclusions

In conclusion, INY, when used as adjunct to pharmacotherapy, was associated with achievingglycaemic control in type 2 DM patients and reducing the overall need for antidiabetes medications within a relatively short span of time. We need to conduct longer-term studies using more rigorous randomized controlled trial designs to confirm these results.

## Additional files

Additional file 1: Codebook. (TXT 8 kb) Additional file 2: Dataset. (XLS 57 kb)
